# Psychometric evaluation of the Chinese version of advance care planning self-efficacy scale among clinical nurses

**DOI:** 10.1186/s12904-022-01064-6

**Published:** 2022-10-07

**Authors:** Zhen Yang, Huan Wang, Aiping Wang

**Affiliations:** The First Affiliated Hospital of China Medical University, No.155, Nanjing North Street, Heping District, Shenyang, Liaoning Province China

**Keywords:** Advance care planning, Self-efficacy, Clinical nurses, Psychometric validation, Cross-cultural adaptation

## Abstract

**Background:**

Nurses are one of the most critical members of advance care planning (ACP) discussion. The evaluation of ACP self-efficacy is of great significance for developing targeted ACP interventions among clinical nurses and update their professional roles. However, there are few instruments to evaluate their ACP self-efficacy in mainland China. The purpose of this study was to translate the ACP self-efficacy scale into Chinese and evaluate its psychometric properties among clinical nurses.

**Methods:**

A methodological study of the translation and validation of the ACP self-efficacy scale was conducted from January to March 2022. It involved three phases: (1) the translation and revision of the scale; (2) the exploration and evaluation of the item (*n* = 436); (3) the psychometric evaluation of the scale (*n* = 674).

**Results:**

After a rigorous translation and revision, the ACP self-efficacy scale with three dimensions and 16 items was finally formed. In this study, the critical ratios of the item ranged from 8.226 to 17.499, and the item-total correlation coefficients ranged from 0.437 to 0.732, and the factor loadings of the item ranged from 0.638 to 0.882. The content validity index of the scale was 0.946. Supported by the eigenvalues, the three-factor structure explained the cumulative 61.131% of the overall variance. As the results of confirmatory factor analysis, all the recommended fitting indexes were appropriate. The average variance extracted values ranged from 0.570 to 0.756, and the composite reliability values ranged from 0.858 to 0.925. The total Cronbach's α coefficient, split-half reliability coefficient and test–retest reliability coefficient of the scale were 0.896, 0.767 and 0.939, respectively.

**Conclusion:**

The Chinese version of ACP self-efficacy scale was successfully introduced into China, showing good psychometric properties among clinical nurses, and can effectively assess the ACP self-efficacy. Also, the scale can provide nursing educators with a significant strategy to develop ACP educational procedure and post-intervention measures for clinical nurses to improve nurse-led ACP practice.

**Supplementary Information:**

The online version contains supplementary material available at 10.1186/s12904-022-01064-6.

## Introduction

Advance care planning (ACP) refers to a process in which individuals can communicate with their surrogate decision-makers and healthcare providers to express future medical care preferences according to their own experiences or values when they are conscious and have the ability to make decisions [1, 2]. ACP circumvented the limitations of advance directives (ADs) as a written document and more flexibly emphasized the process of multi-party communication rather than signing the specific legal document [3]. ACP aims to assist individuals to express their future medical care preferences to reduce decision-making conflicts between individuals and surrogate decision-makers, and it ensure patients' right to know and independent decision-making about future medical care [4, 5]. More significantly, ACP also provides health care professionals with an effective reference for their clinical ethical decision-making [6].

Clinical nurses are one of the most critical members of ACP discussion, serving as full collaborators, participating in the ACP preparation and implementation [7]. Lam et al. [8] reported that 95% of participants did not agree that ACP was solely the responsibility of doctors, and proposed that the initiator of ACP should be nurses. In recent years, there have been more and more studies proving the effectiveness and acceptability of nurse-led ACP practice [9–11]. In addition, nurses are also information providers and educators in ACP communication, and can provide positive ACP guidance and effective ACP interventions for patients and surrogate decision-makers [12]. Hilgeman et al. [13] proposed the patient-centered ACP education intervention program supported by video, the core of which was to provide patients with information about the options, risks and benefits of medical care procedures to help patients make more informed choices and complete ACP discussions. Therefore, nurse-led ACP practice is appropriate and significant.

ACP self-efficacy was the key predictor for patients and clinical nurses to participate in and benefit from ACP discussions [14–16]. Self-efficacy refers to the degree to which individuals are confident that they can use the skills they possess to perform certain behaviors [17]. Clinical nurses with a low ACP self-efficacy may be reluctant to lead or participate in ACP discussions with patients and surrogate decision-makers, or to expend effort in developing ACP skills and addressing barriers to ACP discussions [18]. Therefore, it may be a key component for overcoming the barriers to ACP discussion to improve ACP self-efficacy among clinical nurses. Given the importance of this issue and the urgency of ACP best practices, Baughman et al. [19] developed and validated a scale to assess ACP self-efficacy (ACP-SE) among healthcare providers. The ACP-SE scale had a single dimension with 17 items that explained 58.38% of the total variation and satisfactory reliability and validity [19]. Subsequently, the ACP-SE scale was translated into Spanish and validated its psychometric properties in primary care providers and social workers [20]. To our knowledge, the Chinese version of the ACP-SE scale has not yet been developed. Based on the importance of nurse-led ACP practice, a validated Chinese version of the ACP-SE scale is required to evaluate ACP self-efficacy levels and the effectiveness of ACP-related educational intervention programs among clinical nurses.

The aim of our study was to translate the ACP-SE scale into Chinese and evaluate its psychometric properties among clinical nurses. We expect that the Chinese version of the ACP-SE scale has satisfactory reliability and validity, and the scale can offer an effective strategy for nursing educators to develop educational interventions to improve the ACP self-efficacy of clinical nurses.

## Methods

### Participants

We recruited eligible clinical nurses by convenience sampling in Shenyang and Jinzhou, China, from January to March 2022. Inclusion criteria were registered nurses who have at least one year clinical work experience and gave informed consent. Exclusion criteria were clinical nurses from non-clinical departments and non-Chinese nationalities. The sample size was determined by the general guideline of factor analysis, that is, at least 10 participants responded to each item, and the 20% sample loss rate should also be considered [21]. In our study, the translated scale consists of 16 items, and at least 200 participants were needed, but a large sample is desirable [22]. According to the existing conditions, a total of 436 clinical nurses (for item analysis) and 674 clinical nurses (for psychometric evaluation) were finally recruited.

### Design

**Fig. 1:**
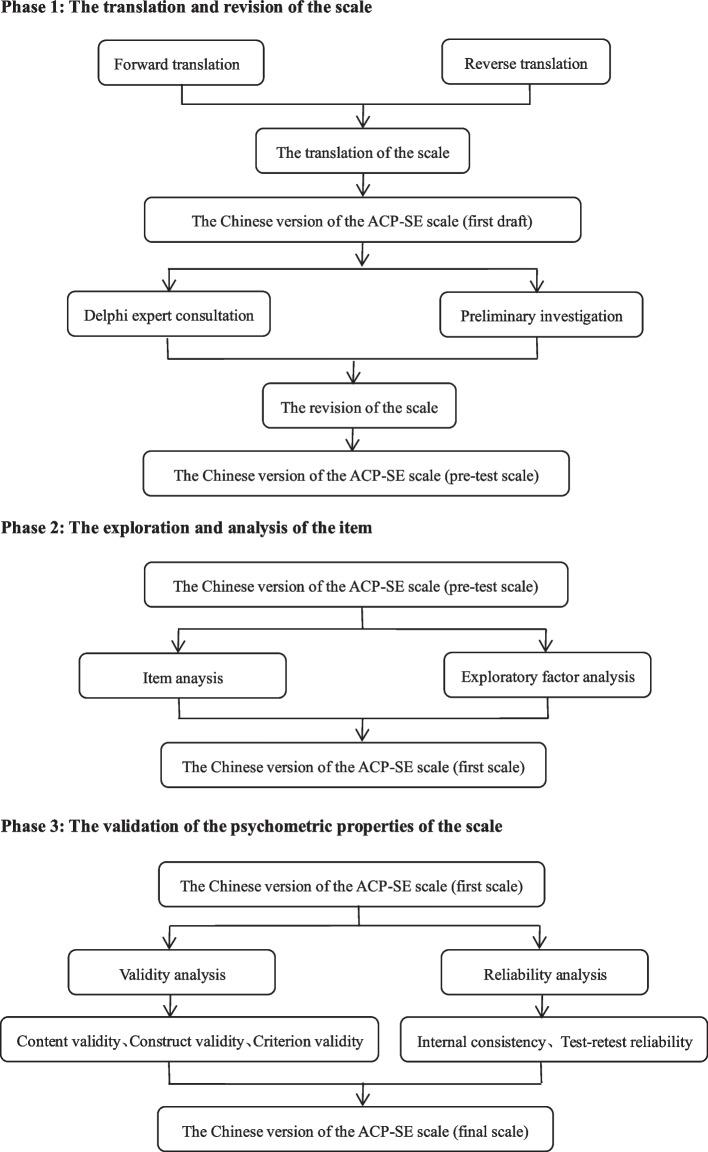
The development procedure of the Chinese version of ACP-SE scale

A methodological study with three phases: (1) the translation and revision of the scale; (2) the exploration and evaluation of the item (*n* = 436); (3) the psychometric evaluation of the scale (*n* = 674). In addition, the samples involved in the third phase were randomly divided into two groups, one for exploratory factor analysis (EFA, *n* = 337) and the other for confirmatory factor analysis (CFA, *n* = 337). The flowchart is shown in Fig. 1.

### The translation and revision of the scale

The Brislin double back-translation method was adopted to translate the ACP-SE scale [23]. First, two Chinese professors (one had experience of studying abroad, the other majored in English) translated the ACP-SE scale into Chinese, respectively. After they discussed with each other, the forward translation version was formed. Then, two foreign teachers translated the forward version into English, respectively. After they discussed with each other, the reverse translation version was formed. Finally, the reverse translation version and the original scale were compared and discussed by the above four translators and the research team to ensure the consistency of semantics and context to the greatest extent. The draft of the Chinese version of the ACP-SE scale was finally formed.

A total of seven experts were invited to revise the draft of the Chinese version of the ACP-SE scale by the Delphi expert consultation method [24]. Inclusion criteria of experts were as follows: (1) at least six years of hospice care study; (2) at least master degree; (3) at least intermediate title; (4) voluntary participants in the study. In this study, the seven expert members included three males and four females with the following credentials: working years (10.43 ± 2.99); four masters, three doctors; all held senior titles. After the cross-cultural adaptation, the draft of the Chinese version of the ACP-SE scale was used for a preliminary investigation among 30 clinical nurses to understand their opinions about the items [25]. Finally, the pre-test Chinese version of the ACP-SE scale was developed.

### The exploration and evaluation of the item

We used the critical ratio method, correlation coefficient method and internal consistency for item analysis to evaluate the suitability for each item. The critical ratio was calculated by t-test for two independent samples (the first 27%, high-score group vs the last 27%, poor-score group) to determine the discrimination of the item. It is generally considered that when the critical ratio of each item ≥ 3 (*P* < 0.05), the item has satisfactory discrimination [26]. The item-total correlation coefficient was calculated to determine the homogeneity of the item, and the item-total correlation coefficient ≥ 0.4 indicates that the item has the appropriate homogeneity [26]. The Cronbach's α coefficient after deleting each item was calculated to determine the quality of the item. We require that the Cronbach's α coefficient of the scale would not increase if the item was deleted [26]. In addition, the preliminary EFA was completed to explore the factor loadings to assess the stability, and the recommended factor loading for each item should be above 0.40 and there was no cross loading. If the above requirements were not met, the corresponding item will be deleted [27].

### The psychometric evaluation of the scale

We invited eligible seven experts to appraise the content validity of the Chinese version of the ACP-SE scale. The Likert four-point scoring system, from one point (irrelevant) to four point (very relevant), was adopted to gather responses from experts. The content validity index of the item (I-CVI) is the ratio of the number of experts giving three or four points to the total number of experts participating in this evaluation. The content validity index of the scale (S-CVI) is the average of I-CVI of all items in the scale. We require the I-CVI ≥ 0.78 and the S-CVI ≥ 0.90 [28].

EFA with principal axis factoring was completed to explore the underlying factor structure of the scale. The Bartlett’s test of sphericity was significant (*P* < 0.05) and the Kaiser–Meyer–Olkin (KMO) coefficient was > 0.60, indicating the suitability for EFA [29]. The requirements are as follows:(1) the factor loading of the item ≥ 0.4 and there was no cross loading, (2) each extracted common factor contains no less than three items, and (3) the cumulative explanatory variation of all common factors ≥ 40% [30, 31].

The CFA was completed to explore whether the factor model met theoretical expectations. The criteria for fitting indexes are as follows: (1) the chi-square degree of freedom (^2^/df) ≤ 3; (2) the root mean square error of approximation (RMSEA) ≤ 0.05; (3) the goodness-of-fit index (GFI), the adjusted goodness-of-fit index (AGFI), the tucker lewis index (TLI), the comparative fit index (CFI), and the incremental fit index (IFI) ≥ 0.9; (4) the parsimonious goodness-of-fit index (PGFI) and the parsimonious normed-of-fit index (PNFI) ≥ 0.5 [32, 33].

Also, convergent validity and discriminant validity were conducted to assess the construct validity of the scale. The average variance extracted (AVE) value and the composite reliability (CR) value were used for the convergent validity. The AVE value ≥ 0.50 and the CR value ≥ 0.70, which indicate that the scale has appropriate convergent validity [34]. The square root of the AVE value and the correlation coefficient of the factors were calculated to evaluate the discriminant validity. We require that the square root of AVE value should exceed the correlation coefficient between the corresponding factors [34].

The ACP-related attitude is closely related to ACP-SE among clinical nurses [16]. Therefore, the ACP practice preference scale for clinical nurses was adopted as a criterion instrument to appraise the criterion validity of the Chinese version of ACP-SE scale. The correlation coefficient between them was calculated as a reliable index, and the correlation coefficient ≥ 0.7, demonstrating that the Chinese version of the ACP-SE scale has optimal criterion validity [35].

The internal consistency and the test–retest reliability were performed to appraise the reliability of the Chinese version of the ACP-SE scale. In the internal consistency analysis, the Cronbach'sα coefficient and the split-half reliability coefficient were calculated to evaluate the homogeneity of the item. After two weeks, the scale was adopted to remeasure the previously labeled 80 clinical nurses, and the correlation coefficient was calculated to assess the stability of the scale across time. We require that the Cronbach’α coefficient, the split-half reliability coefficient and test–retest reliability coefficient should all be 0.7 or above [36, 37].

### Instruments

#### The general demographic characteristics questionnaire

We developed the general demographic characteristics questionnaire after systematic literature analysis and rigorous team negotiation. The questionnaire included seven variables: age, gender, marital status, education level, working years, department, received relevant training.

#### The advance care planning (ACP) practice preference scale

ACP practice preference scale was previously developed by our team to evaluate ACP practice preference among clinical nurses. The scale consists of three dimensions with 24 items. The Likert five-point scoring system (very disagree to very agree) was adopted to gather responses from clinical nurses. The score ranges from 24 to 120. The higher the score is, the stronger the ACP participation preference of clinical nurses is. In our previous work, this scale was confirmed to have appropriate psychometric properties.

### Data collection

After explaining the purpose and significance of the study, the researchers recruited clinical nurses with the assistance of nursing leaders in two cities, China. In phase one, seven eligible experts received the compressed package consisting of informed consent and expert consultation questionnaire via email, and were told to return within two weeks. The recovery rate of the questionnaire was satisfactory. In phase two, we invited 460 clinical nurses to participate in the survey, and 447 clinical nurses agreed to the invitation and signed the informed consent. 436 questionnaires were retained after the questionnaires with missing data were removed. In phase three, we recruited 700 clinical nurses to participate in the survey, and 682 clinical nurses agreed to participate in the study. After deleting the questionnaires with missing data, 674 questionnaires were finally retained. It takes six to eight minutes to complete the questionnaire.

### Data analysis

SPSS 26.0 and AMOS 18.0 were used to complete statistical analysis. Frequency and composition ratios were adopted to describe the general demographic characteristics of clinical nurses. Item analysis was completed to evaluate the quality of the items, and the Delphi survey was adopted to assess the content validity of the scale. EFA with principal axis factoring was completed to explore the underlying factor structure of the scale. Also, the structural equation model (SEM) with maximum likelihood was completed to validate the consistency between the underlying factor structure and the theoretical expectation. The internal consistency analysis and test–retest reliability analysis were conducted to assess the homogeneity and stability of the scale.

All participants signed informed consent and filled in the questionnaire anonymously after being informed of the purpose, significance, voluntary and anonymous nature of the study. All methods and contents of this study were performed in accordance with the Declaration of Helsinki and approved by the Ethics Review Committee of the First Affiliated Hospital of China Medical University (AF-SOP-07–1. 1–01).

## Results

### The general demographic characteristics

In phase two, we finally recruited 436 clinical nurses for the item analysis, and the sample consists of 126 males and 310 females with an average age of (29.22 ± 6.87). In phase three, a total of 337 valid questionnaires composed of 129 males and 208 females with an average age of (29.73 ± 8.07) contributed to the EFA. A total of 337 valid questionnaires, including 135 males and 202 females with an average age of (29.45 ± 7.50) contributed to CFA. More details are showed in Table 1.

**Table 1:** Frequency distribution of demographic characteristics

Characteristics	Phase 2: participants in the item analysis		Stage 3: participants in the EFA		Stage 3: participants in the CFA	
Gender						
Men	126	28.9	129	38.3	135	40.1
Women	310	71.1	208	61.7	202	59.9
Age						
< 25	163	37.4	133	39.5	123	36.5
25–35	167	38.3	107	31.8	118	35.0
> 35	106	24.3	97	28.8	96	28.5
Marital status						
Unmarried	202	46.3	165	49.0	121	35.9
Married	150	34.4	119	35.3	117	34.7
Divorced/Widow	84	19.3	53	15.7	99	29.4
Education levela						
Junior college	108	24.8	80	23.7	84	24.9
Bachelor's degree	206	47.2	147	43.6	162	48.1
Master's degree	122	28.0	110	32.6	91	27.0
Working years						
< 8	204	46.8	147	43.6	152	45.1
8–15	144	33.0	101	30.0	108	32.1
> 15	88	20.2	89	26.4	77	23.8
Department						
Oncology department	92	21.1	94	27.9	87	25.8
ICU, ED, Operating room	127	29.1	102	30.3	105	31.2
Other departments	217	49.8	141	41.8	145	43.0
Received ACP training						
No	276	63.3	220	65.3	212	62.9
Yes	160	36.7	117	34.7	125	37.1

*ED* Emergency department, CFA Confirmatory factor analysis, *EFA* Exploratory factor analysis

### The translation and revision of the scale

After the rigorous translation, we invited seven hospice care experts to revise the draft of the Chinese version of the ACP-SE scale with 17 items. As a result of Delphi survey, the recovery rate of experts consultation questionnaire, the authority coefficient of experts and the Kendall’s concordance coefficient were 1.000, 0.893 and 0.617 respectively. Due to similar statements, we merged "Ensure that patient’s treatment preferences will be honored at your facility" and "Ensure that patient’s treatment preferences will be honored at a hospital if patient is hospitalized" into "Ensure that patient’s care preferences will be honored at your work". In addition, four items were revised due to the stronger pertinence for doctors rather than nurses. For example, "treatment goals and plans" was revised to "care goals and plans", and "life-sustaining treatments" was revised to "life-sustaining care schemes". After a preliminary survey using the revised draft of the Chinese version of the ACP-SE scale, all 30 clinical nurses reported that each item of the scale was easy to understand and answer. The Chinese pretest version of ACP-SE scale with 16 items was finally developed.

### The exploration and analysis of the item

The quality of the item was estimated by the item analysis with the critical ratio, the item-scale correlation coefficient and the Cronbach's α coefficient. In this study, critical ratios of the items ranged from 8.226 to 17.499 (*P* < 0.001), and item-total correlation coefficients ranged from 0.437 to 0.732 (*P* < 0.001). The Cronbach's α coefficient did not increase after each item was deleted. A preliminary EFA was completed to explore the factor loading of each item to assess the stability of the item. As a result of the preliminary EFA, the recommended factor loadings ranged from 0.638 to 0.882 and there was no cross loading. The detailed information is shown in Table 2.

**Table 2:** Item analysis of the scale

Item	*t*-Value	*p*	Factor loading	Cronbach’s α if item deleted	Corrected item‐total correlation coefficients
1	13.152	< 0.001	0.826	0.872 (↓)	0.602^a^
2	8.845	< 0.001	0.790	0.877 (↓)	0.482^a^
3	14.247	< 0.001	0.828	0.872 (↓)	0.602^a^
4	14.414	< 0.001	0.864	0.870 (↓)	0.644^a^
5	11.624	< 0.001	0.869	0.874 (↓)	0.541^a^
6	12.166	< 0.001	0.850	0.872 (↓)	0.584^a^
7	10.617	< 0.001	0.811	0.875 (↓)	0.520^a^
8	13.101	< 0.001	0.847	0.872 (↓)	0.596^a^
9	13.657	< 0.001	0.826	0.871 (↓)	0.616^a^
10	8.226	< 0.001	0.662	0.878 (↓)	0.437^a^
11	17.211	< 0.001	0.882	0.865 (↓)	0.731^a^
12	13.935	< 0.001	0.639	0.871 (↓)	0.608^a^
13	17.499	< 0.001	0.882	0.865 (↓)	0.732^a^
14	17.029	< 0.001	0.638	0.868 (↓)	0.669^a^
15	13.172	< 0.001	0.837	0.872 (↓)	0.607^a^
16	12.533	< 0.001	0.683	0.875 (↓)	0.561^a^

^a^indicates significance *p* < 0.01; "↓" indicates that once the item is deleted, the Cronbach's α decreases

### The psychometric evaluation of the scale

#### Content validity

Seven qualified hospice care experts were invited to assess the I-CVI and S-CVI. As a result of Delphi expert consultation, the expert response rate was 100%, and I-CVI ranged from 0.857 to 1.000, and S-CVI was 0.946.

#### Construct validity

**Fig. 2:**
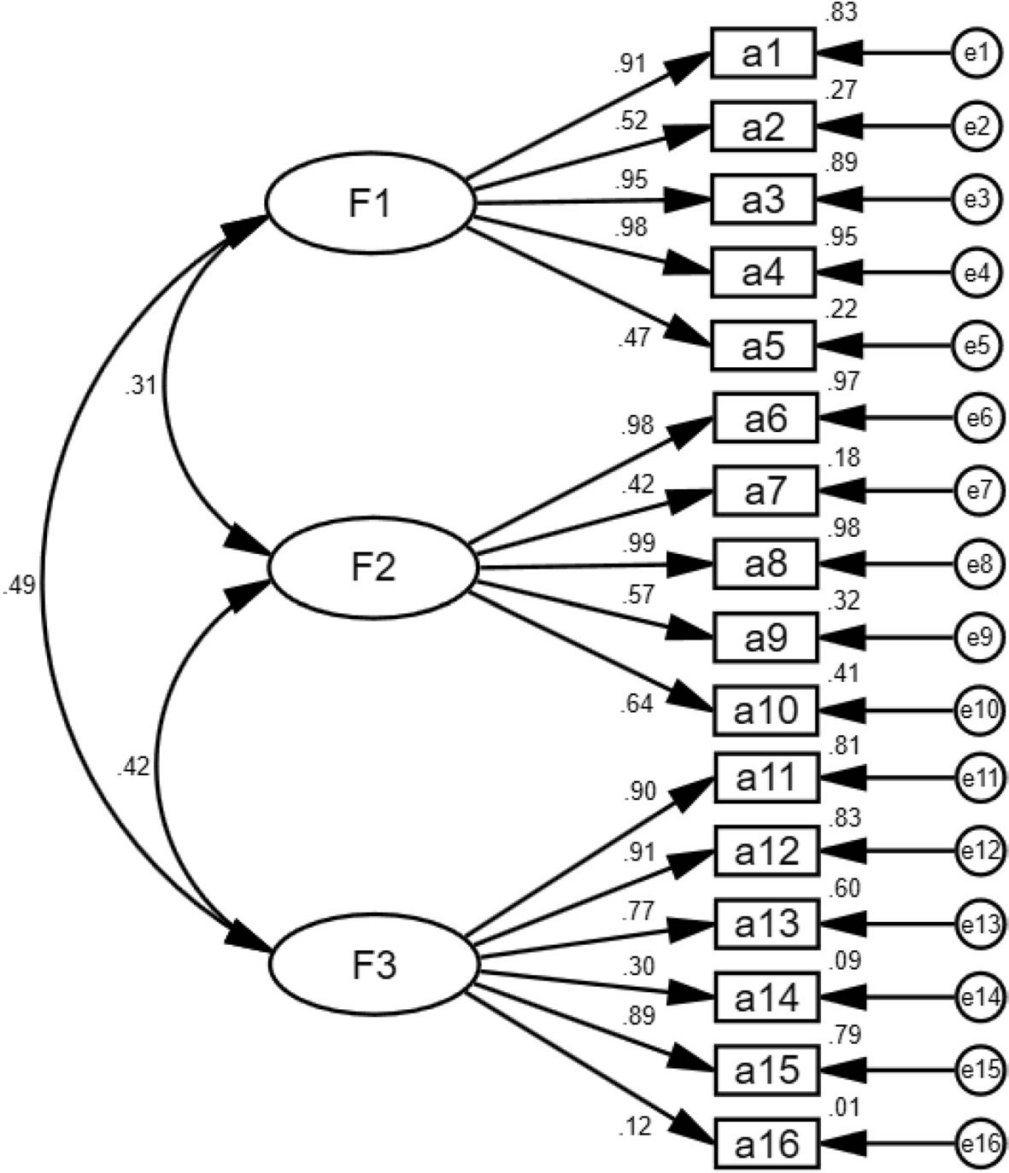
Standardized three-factor model of the Chinese version of ACP-SE scale

In EFA, the KMO value was 0.883, and the Bartlett sphericity test was significant (^2^ = 4571.576, *P* < 0.001). The three-factor model explained 61.131% of the total variation with eigenvalues > 1 (Table 3). Based on the meaning expressed by the item, the three factors were named preference discussion and evaluation, information guidance and disclosure, and content evaluation and determination, respectively. In CFA, the SEM with the maximum likelihood method was adopted to fit the three-factor model (Fig. 2). As the results of CFA, ^2^/df = 2.046, GFI = 0.929, AGFI = 0.904, RMSEA = 0.046, TLI = 0.969, CFI = 0.974, IFI = 0.974, PGFI = 0.690, PNFI = 0.800. The selected fitting indexes showed that the three-factor model has appropriate fitting. However, unlike the results of EFA, the results of CFA showed that factors 14 and 16 were weak and should be removed from the scale, and the Chinese version of ACP-SE scale with 3 dimensions and 14 items was finally developed. In convergent validity analysis, the AVE values ranged from 0.570 to 0.756, and CR values ranged from 0.858 to 0.925. In discriminant validity analysis, the square root values of AVE ranged from 0.755 to 0.869, which were all greater than the correlation coefficient between the corresponding factors (Table 4).

**Table 3:** Pattern matrix of the scale after the factor analysis

Item	Factor 1	Factor 2	Factor 3
Find time to discuss prognosis, preference and care plan with patients	0.190	**0.870**	0.193
Discuss and negotiate individualized care goals and plans with the patient	0.149	**0.561**	0.115
Discuss with the patient how to complete the living will	0.125	**0.693**	0.106
Respond compassionately to the concerns of patients and families	0.249	**0.903**	0.196
Reassess the patient’s wishes when a shift in care goals is needed	0.223	**0.939**	0.208
Provide the information and guidance to help the patient make decisions	0.222	0.183	**0.928**
Describe the pros and cons of different life-sustaining care schemes	0.196	0.206	**0.550**
Discuss the existing uncertainty openly with patients	0.211	0.183	**0.944**
Educate patient and clarify any misconceptions on the disease or prognosis	0.306	0.145	**0.563**
Deliver "bad news" to patients and their families	0.129	0.108	**0.619**
Determine how much the patient wants to know about the prognosis	**0.802**	0.187	0.190
Determine the level of involvement the patient wants in decision-making	**0.828**	0.212	0.136
Determine the surrogate decision-maker the patient wants	**0.832**	0.219	0.207
Determine the patient's specific wishes for the type of care	**0.537**	0.107	0.125
Determine when there should be a shift in care goals	**0.886**	0.162	0.218
Ensure that patient’s care preferences will be honored at your work	**0.536**	0.148	0.216

**Table 4:** Convergent validity and discriminant validity of the scale

Factors	Correlation between factors			AVE	Sqrt (AVE)	CR
	**Factor 1**	**Factor 2**	**Factor 3**			
Factor 1	1			0.636	0.797	0.889
Factor 2	0.313	1		0.570	0.755	0.858
Factor 3	0.460	0.410	1	0.756	0.869	0.925

*AVE* Average variance extracted, *CR* Composite reliability

#### Criterion validity

The ACP practice preference scale was adopted as the criterion instrument to measure the criterion validity of the Chinese version of the ACP-SE scale. As a result of the correlation analysis, the ACP-SE was highly positively correlated with the ACP practice preference, and the correlation coefficient was 0.889 (*P* < 0.001).

### Internal consistency and test–retest reliability

The homogeneity and stability of the Chinese version of the ACP-SE scale were evaluated by the internal consistency and the test–retest reliability. As a result of reliability analysis, the total Cronbach's α coefficient of the scale was 0.896 with three dimensions of 0.885, 0.849 and 0.927, respectively. The total split-half reliability coefficient of the scale was 0.767 with three dimensions of 0.912, 0.827 and 0.917, respectively. The total test–retest reliability coefficient of the scale was 0.939 with three dimensions of 0.924, 0.835, 0.849, respectively.

## Discussion

There are lacks of instruments to assess ACP-SE among clinical nurses in China. This study translated the ACP-SE scale into Chinese for the first time [23], and validated its psychometric properties among clinical nurses by factor analysis [30]. The Chinese version of the ACP-SE scale with appropriate reliability and validity could be considered as an effective instrument for assessing ACP-SE among clinical nurses. Moreover, it can also provide nursing educators with a significant strategy to develop ACP education interventions for clinical nurses and to assess ACP-SE after intervention based on the items and dimensions to improve their ACP participation and the competence of hospice care.

### The practicality of the scale

The original version of the ACP-SE scale consists of a single dimension with seventeen items [19]. In this study, after rigorous translation and cross-cultural adaptation [24], the Chinese version of the ACP-SE scale with the three-factor structure was developed. Based on the connotation reflected by the item, the common factors were assigned to different aspects of the nurse-led ACP practice, which were named as preference discussion and evaluation, information guidance and disclosure, and content evaluation and determination respectively. Compared with the original version [19], the Chinese version of the ACP-SE scale with a three-factor structure further detailed and clarified the different aspects of ACP-SE in clinical nurses, which can measure the shortcomings of clinical nurses in a certain aspect of ACP practice, and also pointed out the direction of the targeted intervention. Therefore, the Chinese version of the ACP-SE scale had a certain practicality, that was, it could comprehensively assess the ACP-SE level of clinical nurses.

### The scientificity of the scale

Based on strict evaluation and revision of the items, the Chinese version of the ACP-SE scale with three dimensions and sixteen items was developed. In item analysis, the critical ratios of the items met the reference standard [26], and there was a moderate to high correlation between item-total scores. In addition, when the item was deleted, the Cronbach's α coefficient on the scale did not increase. Therefore, the above information proved that the items have optimal applicability [26, 27].

In content validity, I-CVI and S-CVI were superior to the standard value, supporting the suitable content validity of the scale [28]. The total variation is well explained by the three-factor structure extracted from EFA. Also, the expected theoretical model was further confirmed by CFA, and the model fitting indexes were satisfactory [32, 33]. Moreover, the AVE and CR values were appropriate, and the square root of AVE values was greater than the correlation coefficient between the corresponding factors, which showed that the scale has good convergent validity and discrimination validity [34]. The above information strongly proves that the construct validity of the scale is appropriate. In calibration validity, the high correlation between the ACP-SE and the ACP participation preference also demonstrated that the scale has suitable calibration validity.

In reliability analysis, the Cronbach's α coefficient and the split-half reliability coefficient of the scale were both above the reference value [36] but slightly lower than the original version and the Spanish version [19, 20], which indicated the scale has an optimal internal consistency. Moreover, the previously labeled nurses were remeasured two weeks later, and the test–retest reliability coefficient reached an acceptable range [36], indicating that the scale has measurement stability across time [38].

### Limitations

We consider some limitations that need to be paid attention to and discussed in our study. First, a large multi-center sample is worth considering to improve the sample representativeness and explore the cultural differences represented by different regions, although the sample size of the study met the statistical requirements. Second, bias from the nature of convenience sampling is inevitable.Based on this, the rigorous data collection procedure were considered to reduce selection bias, and the 20% sample loss rate was also considered in our study. Finally, as the focus of our future work, the Chinese version of the ACP-SE scale was used to evaluate its predictive effectiveness.

## Conclusion

The Chinese version of ACP-SE scale was successfully introduced into China. It showed good psychometric properties among clinical nurses and can effectively assess the ACP-SE. It can also provide a significant strategy for nursing educators to develop ACP education interventions and post-intervention measures among clinical nurses to improve their ACP participation.

## Supplementary Information

**Additional file 1.**

## Data Availability

The datasets used and/or analysed during the current study are available from the corresponding author on reasonable request.

## Abbreviations

ACP: Advance Care Planning
ADs: Advance Directives
ACP-SE: ACP Self-Efficacy
SEM: Structural Equation Model
I-CVI: Content Validity Index of the Item
S-CVI: Content Validity Index of the Scale
KMO: Kaiser–Meyer–Olkin; EFA: Exploratory Factor Analysis
CFA: Confirmatory Factor Analysis
χ^2^/df: Chi-Square Degree of Freedom
RMSEA: Root Mean Square Error of Approximation
GFI: Goodness-of-Fit Index
AGFI: Adjusted Goodness-of-Fit Index
TLI: Tucker Lewis Index
CFI: Comparative Fit Index
IFI: Incremental Fit Index
PGFI: Parsimonious Goodness-of-Fit Index
PNFI: Parsimonious Normed-of-Fit Index
AVE: Average Variance Extracted
CR: Composite Reliability
