# Supplementary material for: Psychometric evaluation of the Chinese version of advance care planning self-efficacy scale among clinical nurses
Source: BMC Palliat Care. 2022 Oct 7;21:175. doi: 10.1186/s12904-022-01064-6 (PMC9541061; doi:10.1186/s12904-022-01064-6)
Supplement: Supplementary file 1 — Additional file 1. [file 12904_2022_1064_MOESM1_ESM.docx]

**The Chinese Version of Advance Care Planning Self-Efficacy Scale (English Language)**

| **Preference Discussion and Evaluation** | Very unconfident | Unconfident | Unsure | Confident | Very confident |
| --- | --- | --- | --- | --- | --- |
| 1. Find time to discuss prognosis, preference and care plan with patients. | 1 | 2 | 3 | 4 | 5 |
| 2. Discuss and negotiate individualized care goals and plans with the patient. | 1 | 2 | 3 | 4 | 5 |
| 3. Discuss with the patient how to complete the living will. | 1 | 2 | 3 | 4 | 5 |
| 4. Respond compassionately to the concerns of patients and families. | 1 | 2 | 3 | 4 | 5 |
| 5. Reassess the patient’s wishes when a shift in care goals is needed. | 1 | 2 | 3 | 4 | 5 |
| **Information Guidance and Disclosure** | Very unconfident | Unconfident | Unsure | Confident | Very confident |
| 1. Provide the information and guidance to help the patient make decisions | 1 | 2 | 3 | 4 | 5 |
| 2. Describe the pros and cons of different life-sustaining care schemes. | 1 | 2 | 3 | 4 | 5 |
| 3. Discuss the existing uncertainty openly with patients. | 1 | 2 | 3 | 4 | 5 |
| 4. Educate patient and clarify any misconceptions on the disease or prognosis. | 1 | 2 | 3 | 4 | 5 |
| 5. Deliver "bad news" to patients and their families. | 1 | 2 | 3 | 4 | 5 |
| **Content Evaluation and Determination** | Very unconfident | Unconfident | Unsure | Confident | Very confident |
| 1. Determine how much the patient wants to know about the prognosis. | 1 | 2 | 3 | 4 | 5 |
| 2. Determine the level of involvement the patient wants in decision-making. | 1 | 2 | 3 | 4 | 5 |
| 3. Determine the surrogate decision-maker the patient wants. | 1 | 2 | 3 | 4 | 5 |
| 4. Determine the patient's specific wishes for the type of care. | 1 | 2 | 3 | 4 | 5 |
| Involve patients in advance care planning conversations.* | 1 | 2 | 3 | 4 | 5 |

**Note：*** is a general item that includes all advance care planning and not part of the scale. It can be used for comparison to the scale.
